# Tetracycline-Inactivating Enzymes

**DOI:** 10.3389/fmicb.2018.01058

**Published:** 2018-05-30

**Authors:** Jana L. Markley, Timothy A. Wencewicz

**Affiliations:** Department of Chemistry, Washington University in St. Louis, St. Louis, MO, United States

**Keywords:** tetracycline destructases, enzymatic antibiotic inactivation, antibiotic adjuvants, tetracyclines, antibiotic resistance, flavin monooxygenase

## Abstract

Tetracyclines have been foundational antibacterial agents for more than 70 years. Renewed interest in tetracycline antibiotics is being driven by advancements in tetracycline synthesis and strategic scaffold modifications designed to overcome established clinical resistance mechanisms including efflux and ribosome protection. Emerging new resistance mechanisms, including enzymatic antibiotic inactivation, threaten recent progress on bringing these next-generation tetracyclines to the clinic. Here we review the current state of knowledge on the structure, mechanism, and inhibition of tetracycline-inactivating enzymes.

## Introduction

### Tetracycline Antibiotics

**FIGURE 1:**
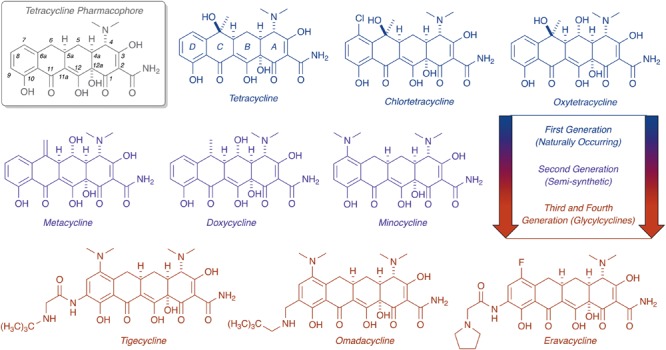
Evolution of the tetracycline scaffold. 6-Deoxy-6-demethyltetracycline represents the minimum tetracycline pharmacophore required for inhibition of the ribosome. Tetracycline (first reported in 1953), CTc (first reported in 1948), and oxytetracycline (first reported in 1950) represent first generation structures. Metacycline (first reported in 1962), doxycycline (first reported in 1967), and minocycline (first reported in 1961) represent second generation structures. Tigecycline (first reported in 1993) is the only FDA-approved third generation structure, while omadacycline (first reported in 2013) and eravacycline (first reported in 2013) are fourth generation molecules currently in phase III clinical trials.

Tetracycline antibiotics were discovered in the 1940s and found widespread clinical use shortly thereafter (Duggar, 1948; Finlay et al., 1950; King et al., 1950; Roberts, 1996; Nelson and Levy, 2011). Naturally occurring tetracyclines are highly oxidized, type II polyketides composed of a linear fused tetracyclic scaffold with rings designated A, B, C, D (**Figure 1**; Stephens et al., 1952; Chopra and Roberts, 2001). Tetracyclines inhibit bacterial protein synthesis by binding to the 16S rRNA of the 30S bacterial ribosome subunit, preventing accommodation of incoming aminoacyl-tRNAs at the acceptor site (A-site) (Brodersen et al., 2000; Wilson, 2009). Tetracyclines make sequence-independent contacts with sugar phosphates in the primary binding site between h31 and h34. Both synthetic and semisynthetic tetracyclines have found clinical use as low cost, broad-spectrum, and orally available antimicrobial agents. The minimum active pharmacophore for bacterial ribosome inhibition is 6-deoxy-6-demethyltetracycline (McCormick et al., 1960; Chopra and Roberts, 2001). Chemical modification of positions 5–9 is well tolerated and can improve ribosome affinity, as is the case for the first and second generation scaffolds CTc and doxycycline. Modification of positions 1–4 and 10–12 strongly attenuates the antibacterial activity. The 1,3-diketone group at carbons 11 and 12 (pKa ∼7) chelates Mg^2+^ (Stephens et al., 1956; Jin et al., 2007). The tetracycline–Mg complex is the biologically active form that permeates the bacterial cell envelope (Schnappinger and Hillen, 1996) and binds to bacterial ribosomes (Jenner et al., 2013), transcription factors (Hinrichs et al., 1994), and aptamers (Xiao et al., 2008).

Chemical modification of the tetracycline scaffold has preserved this important class of antibiotics for >70 years against continuous waves of resistance determinants (Charest et al., 2005; Liu and Myers, 2016; Sun and Xiao, 2017). The unique 3D chemical shape of tetracycline arises from a bend in the structure at the A,B-ring juncture, and this seems to be a distinguishing feature from other tetracyclic polyketides that impart selectivity for ribosome binding (Brodersen et al., 2000; Stepanek et al., 2016). The D-ring of tetracyclines has proven to be robust toward semi-synthetic modifications, as highlighted by the bulky *N*-*t*-butyl-glycylamide side chain of the third generation antibiotic tigecycline, which plays a dual role in overcoming resistance and increasing affinity for the 30S ribosomal subunit (Jenner et al., 2013). Access to fully synthetic tetracyclines, including fourth generation compounds eravacycline (Ronn et al., 2013; Zhanel et al., 2016) and omadacycline (Tanaka et al., 2016) – both currently in phase III clinical trials – has rejuvenated clinical prospects for this drug class (Liu and Myers, 2016; Sun and Xiao, 2017). With the approval of next-generation tetracyclines on the horizon, new mechanisms of tetracycline resistance are certain to emerge as clinical use increases. Our ability to manage emerging resistance is critical to ensure future utility of tetracycline antibiotics and prevent a public health care crisis (Brown and Wright, 2016).

### Tetracycline Resistance

**FIGURE 2:**
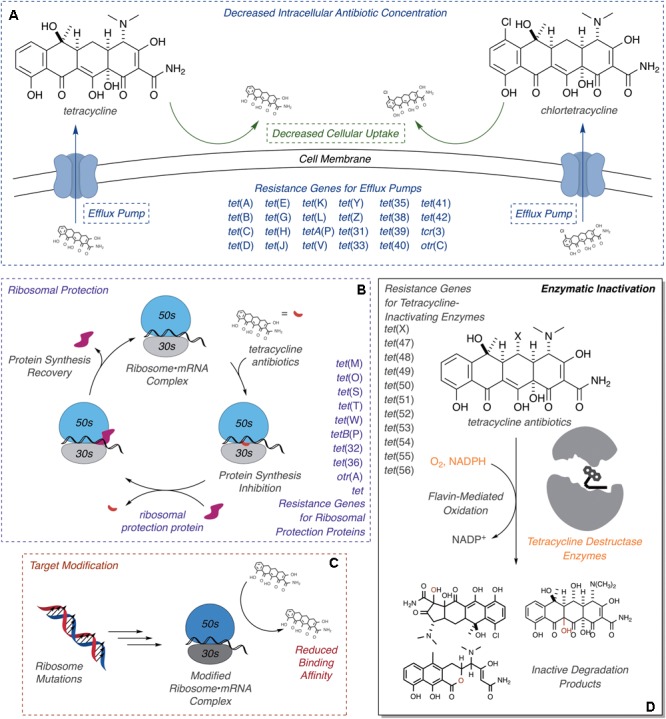
Molecular mechanisms of tetracycline resistance. **(A)** Efflux, exclusion, **(B)** ribosome protection, **(C)** ribosome modification, and **(D)** enzymatic inactivation. Documented ARGs associated with each type of tetracycline resistance are provided.

Resistance to tetracycline antibiotics was observed from the very start of clinical use (Schiott and Stenderup, 1954; Roberts, 1996). Despite widespread clinical resistance, tetracyclines continue to be important agents for treating a variety of human infections caused by Gram-negative and Gram-positive bacterial pathogens, along with atypical pathogens including mycoplasmas, nematodes, and parasitic protozoans (Chopra and Roberts, 2001). Tetracyclines are also widely used in veterinary medicine and agricultural applications, including crop protection and intensive animal farming, which has contributed to the widespread dissemination of tetracycline resistance (Thaker et al., 2010; Surette and Wright, 2017). Molecular mechanisms of tetracycline resistance include efflux (Izaki and Arima, 1963; Levy and McMurry, 1978; Kaneko et al., 1985), ribosome protection proteins (Burdett et al., 1982; Burdett, 1986, 1991, 1996), reduced permeability (Cohen et al., 1988), ribosome mutation (Ross et al., 1998), and enzymatic inactivation (**Figure 2**; Yang et al., 2004; Nguyen et al., 2014). Efflux pumps and ribosome protection proteins are the most common types of clinical resistance to tetracyclines and have been found in most human pathogens (Connell et al., 2003; Piddock, 2006; Thaker et al., 2010). Seven groups of efflux pumps have been identified that confer tetracycline resistance by decreasing the effective intracellular antibiotic concentration, with most members falling into the major facilitator superfamily (Guillaume et al., 2004; Piddock, 2006). Ribosome protection proteins are GTPases with homology to elongation factors that bind the ribosome analogously to elongation factors and chase bound tetracycline from the 30S ribosomal subunit (Connell et al., 2003; Jenner et al., 2013). Reduced drug permeability is achieved through morphological changes and the modification or reduced expression of porins and likely contributes to clinical tetracycline resistance (Cohen et al., 1988; Schnappinger and Hillen, 1996; Olesky et al., 2006; Justice et al., 2008). Ribosomal mutations are uncommon in clinical resistance to tetracyclines, probably due to the sequence-independent binding mode of tetracycline to the 30S ribosomal subunit (Brodersen et al., 2000); still, some resistance-conferring mutations and deletions around the tetracycline-binding site have been reported (Ross et al., 1998; Gerritis et al., 2002; Trieber and Taylor, 2002). Some clinical isolates of *Helicobacter pylori* (Nonaka et al., 2005) and *Propionibacterium acnes* (Ross et al., 1998) carry point mutations in the 16S ribosome that confer tetracycline resistance, presumably through reduced tetracycline binding affinity. These ribosome mutations also confer tetracycline resistance in laboratory strains of *Escherichia coli* (Cocozaki et al., 2016). Similar resistance to tigecycline in *S. pneumonia*, arising from point mutations in ribosomal proteins and rRNA, has been introduced in the laboratory via successive passaging (Lupien et al., 2015). A more obscure resistance mechanism involves activation of Mg^2+^-dependent purine nucleotide biosynthesis via expression of the *tet34* gene product, a predicted xanthine–guanine phosphoribosyltransferase, which attenuates tetracycline antibacterial activity presumably by increasing the pool of GTP available to elongation factors to accelerate binding of aminoacyl-tRNAs to the 30S ribosomal subunit (Nonaka and Suzuki, 2002; Kim et al., 2003).

Third (tigecycline) and fourth generation (eravacycline and omadacycline) tetracyclines are known to overcome resistance via efflux and ribosome protection (Jenner et al., 2013; Zhanel et al., 2016; Tanaka et al., 2016). However, enzymatic inactivation has emerged as a new concern for these next-generation tetracyclines (Moore et al., 2005; Grossman et al., 2012, 2017). A family of FMOs, the tetracycline destructases (Forsberg et al., 2015), has been shown to selectively oxidize tetracyclines leading to covalent destruction of the antibiotic scaffold (Yang et al., 2004). Unlike efflux, exclusion, ribosome protection, and ribosome modification, enzymatic inactivation permanently eliminates the tetracycline antibiotic challenge by decreasing intracellular and extracellular antibiotic concentrations (Davies, 1994; Wright, 2005). The clinical impact of enzymatic antibiotic inactivation can be devastating, as documented by the spread of broad-spectrum beta-lactamases across the globe (Bush and Jacoby, 2010; Brandt et al., 2017). The goal of this review is to highlight recent advances involving the structure, mechanism, and inhibition of tetracycline destructases to bring awareness and inspire solutions for this emerging type of tetracycline resistance.

## Tetracycline Destructases

### Antibiotic Destructases

The tetracycline destructases are part of a broadly defined family of enzymes, which we are calling the antibiotic destructases, that inactivate antibiotics via a wide variety of covalent modifications to the antibiotic scaffold (Davies, 1994; Wright, 2005). Antibiotic destructases are named to reflect the enzymatic activity associated with covalent modification of antibiotic scaffolds that permanently destroys antimicrobial activity and imparts resistance to producing microbes. Antibiotic destructases differ from xenobiotic modifying metabolic enzymes in regulation, catalytic efficiency, rate, and substrate specificity. Xenobiotic modifying enzymes perform housekeeping functions in the host, primarily clearance, and detoxification of xenobiotics (Krueger and Williams, 2005). The primary function of antibiotic destructases is gain of resistance. Thus, xenobiotic modifying enzymes tend to be broad in substrate scope at the cost of catalytic efficiency, while antibiotic destructases tend to be narrower in substrate scope with high specificity and catalytic efficiency toward a particular structural class of antibiotics (Wright, 2005).

Well-known examples of antibiotic destructases include beta-lactamases that hydrolyze the strained 4-membered lactam of beta-lactam antibiotics (Bush and Jacoby, 2010; Brandt et al., 2017), and aminoglycoside-inactivating enzymes including phosphotransferases, acetyltransferases, and adenylyltransferases that modify the free amine and hydroxyl groups of aminoglycoside antibiotics (Ramirez and Tolmasky, 2010). Known classes of antibiotic destructases (antibiotic substrates) include peptidases (bogorol, bacitracin) (Li et al., 2018), hydrolases (beta-lactams, macrolides) (Bush and Jacoby, 2010; Morar et al., 2012), thioltransferases (fosfomycin) (Rife et al., 2002; Thompson et al., 2013), epoxidases (fosfomycin) (Fillgrove et al., 2003), cyclopropanases (colibactin) (Tripathi et al., 2017), acyl transferases (aminoglycosides, chloramphenicol, glufosinate, tabtoxinine-beta-lactam, streptogramin) (Leslie, 1990; Botterman et al., 1991; Sugantino and Roderick, 2002; Ramirez and Tolmasky, 2010; Wencewicz and Walsh, 2012; Favrot et al., 2016), methyl transferases (holomycin) (Li et al., 2012; Warrier et al., 2016), nucleotidylyl transferases (aminoglycosides, lincosamide) (Morar et al., 2009; Ramirez and Tolmasky, 2010), ADP-ribosyltransferases (rifamycins) (Baysarowich et al., 2008), glycosyltransferases (aminoglycosides, rifamycins, macrolides) (Bolam et al., 2007; Ramirez and Tolmasky, 2010; Spanogiannopoulos et al., 2012), phosphotransferases (aminoglycosides, chloramphenicol, rifamycins, macrolides, viomycin) (Thiara and Cundliffe, 1995; Izard and Ellis, 2000; Ramirez and Tolmasky, 2010; Stogios et al., 2016; Fong et al., 2017), lyases (streptogramins) (Korczynska et al., 2007), and oxidoreductases (tetracyclines, rifamycins) (Park et al., 2017; Koteva et al., 2018). As antibiotic prospecting continues, the list of antibiotic destructases is certain to grow (Crofts et al., 2017; Li et al., 2018; Pawlowski et al., 2018).

Unlike other major classes of antibiotic resistance (efflux, exclusion, target modification), covalent inactivation by antibiotic destructases permanently neutralizes the antibiotic challenge and lowers intracellular and extracellular antibiotic concentrations. If antibiotic levels fall below the MIC, then resistance is achieved. Covalent modification of antibiotics can perturb target affinity, block cellular uptake, trigger efflux mechanisms, or lead to decomposition of the antibiotic (Wright, 2005). Genes encoding for antibiotic destructases are often present in operons that are co-transcribed with biosynthetic genes in the antibiotic producing microbe (Li et al., 2018). Co-transcription ensures self-protection during antibiotic biosynthesis (Bolam et al., 2007; Mack et al., 2014). Antibiotic destructases are often transferable through mobilized genetic elements such as plasmids (Davies and Davies, 2010). Once transformed into a host microbial cell, the expression of antibiotic destructases is often inducible and in some cases can be triggered specifically in response to antibiotic challenge (Llarrull et al., 2011). Antibiotic destructases can be excreted to the periplasm or even the extracellular space in order to destroy the antibiotic before it reaches the microbial cell. Resistance caused by antibiotic destructases can be overcome, in theory, by modifying the antibiotic scaffold to evade destructases (Syriopoulou et al., 1981), co-administration of a destructase inhibitor (Drawz and Bonomo, 2010), inhibition of destructase production or localization (Therien et al., 2012), or increasing intracellular concentrations to overcome destructase production (McPherson et al., 2012). Thus far, only modification of the antibiotic scaffold and co-administration of a destructase inhibitor have proven effective for overcoming resistance by antibiotic destructases in clinical infections (Fisher et al., 2005; Drawz and Bonomo, 2010).

Each class of antibiotic destructase represents a distinct chemical mode of antibiotic inactivation with the evolutionary potential to broaden or narrow substrate discrimination (Pawlowski et al., 2018). The evolutionary landscape leans heavily in favor of optimizing resistance enzymes due to the widespread selective pressure applied by broad-spectrum antibiotics. To prepare and respond to the emergence of antibiotic destructases, a thorough understanding of the genetic origins, dissemination, structure, and mechanism of the antibiotic destructase must be established. The rise of beta-lactamases in hospital- and community-acquired infections is the historical model for resistance via antibiotic destruction. Continuous innovation around the beta-lactam pharmacophore and co-administration of beta-lactamase inhibitors as adjuvants has maintained the clinical viability of this important antibiotic class (Bush, 2018). Most of the antibiotic-inactivating enzymes cited above do not represent current clinical threats; however, each threatens to emerge pending widespread clinical use of the corresponding antibiotic class. The recent success of fourth generation tetracyclines in advanced clinical trials has raised concerns over selecting for tetracycline destructases that might compromise future clinical use of the entire tetracycline class of antibiotics.

### TetX – The Flagship Tetracycline-Inactivating Enzyme

Enzymatic inactivation of tetracyclines was first proposed as a resistance mechanism in 1984 (Guiney et al., 1984). A plasmid that conferred tetracycline resistance to *E. coli* with a strict requirement for aerobic growth was isolated from the human commensal *Bacteroides fragilis* (Matthews and Guiney, 1986; Park et al., 1987; Speer and Salyers, 1988). Plasmid mapping revealed the presence of a putative tetracycline efflux pump and a gene, *tetX*, encoding for a potentially novel tetracycline resistance enzyme that catalyzes tetracycline degradation (Park and Levy, 1988). Growth of *E. coli* carrying the *tetX* gene on an inducible plasmid yielded a distinctive brown colored growth phenotype, exclusively under aerobic conditions (Speer and Salyers, 1989). Spent media from tetracycline-treated cultures of *E. coli* expressing the *tetX* gene showed decreased tetracycline concentrations and loss of tetracycline activity against wild-type *E. coli*. Cell-free lysates of *E. coli* expressing *tetX* strictly required exogenous NADPH for tetracycline inactivation, consistent with TetX being an NADPH-dependent oxidoreductase (Speer et al., 1991). Two additional variants of the *tetX* gene, *tetX1* and *tetX2*, were later identified in another *Bacteroides* transposon (Whittle et al., 2001).

**FIGURE 3:**
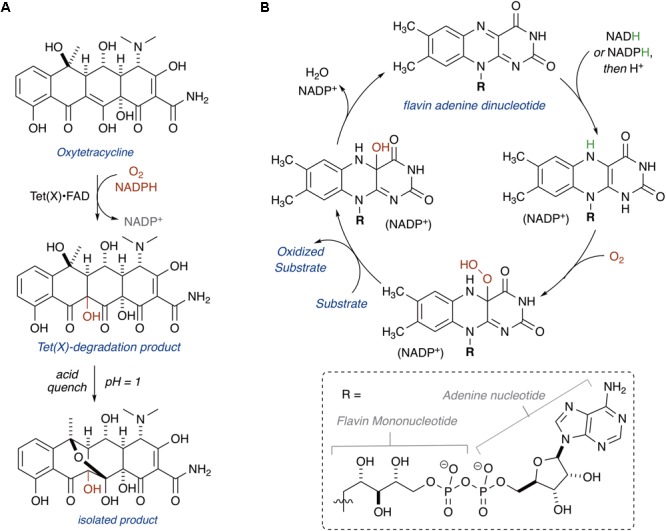
**(A)** Hydroxylation of oxytetracycline by TetX. **(B)** Mechanism of class A FMOs.

In 2004, Wright and coworkers heterologously expressed TetX, TetX1, and TetX2 in *E. coli* and purified the recombinant proteins (Yang et al., 2004). TetX and TetX2 are 99% sequence identical, and both proteins co-purified with a bound flavin cofactor and proved to be active FMOs that degrade tetracyclines. TetX1 is a truncated variant that does not bind flavin and is thus not a true tetracycline resistance enzyme. TetX was shown to inactivate first, second, and third generation tetracyclines including tigecycline (Moore et al., 2005). Oxidation of oxytetracycline by TetX leads to formation of a variety of degradation products, including hydroxylation at C11a, the product of which was isolated and characterized by Wright and coworkers following an acid quench that provided the stabilized cyclic hemiketal (**Figure 3A**; alternate sites of oxidation and mechanistic considerations are discussed below in the section “Mechanisms of Tetracycline Oxidation”) (Yang et al., 2004). Presumably, modification at C11a will attenuate Mg^2+^ chelation and ribosome binding, which are both required for biological activity of oxytetracycline (Schnappinger and Hillen, 1996). Additionally, hydroxylation of C11a destabilizes the tetracycline scaffold leading to complex mixtures of non-enzymatic degradation products (Yang et al., 2004). X-ray crystal structures of TetX bound to 7-CTc, 7-iodotetracycline, minocycline, and tigecycline have been reported and confirmed TetX to be a class A FMO (structures are discussed below in the section “Structural Basis for Tetracycline Inactivation”) (Volkers et al., 2011, 2013). Similar to other class A FMOs, TetX is predicted to utilize NADPH to reduce the flavin cofactor in preparation for subsequent formation of a reactive C4a-peroxyflavin that transfers an electrophilic hydroxyl group to the nucleophilic C11a of the tetracycline enol (**Figure 3B**; van Berkel et al., 2006).

### Expanding the Tetracycline Destructase Family

Mobilization of *tetX* on transposons in *Bacteroides* suggests that dissemination of the tetracycline resistance gene into human pathogens is possible (Whittle et al., 2002). Indeed, in 2013, the *tetX* gene was found in a variety of MDR Gram-negative pathogens (*Enterobacter cloacae, Comamonas testosteroni, E. coli, Klebsiella pneumonia, Delftia acidovorans*, and other members of Enterobacteriaceae and Pseudomonadaceae) isolated from a hospital in Sierra Leone (Leski et al., 2013). Several of the *tetX* harboring pathogens are on the CDC’s list of ESKAPE pathogens (Santajit and Indrawattana, 2016), including *Pseudomonas aeruginosa* and *Acinetobacter baumannii* (Aminov, 2009, 2013; Deng et al., 2014). Although *tetX* has been found in human pathogens, there is yet to be a documented clinical case of tetracycline resistance caused by *tetX* or related genes encoding for tetracycline destructases. The *tetX* gene has also been observed in a variety of environmental bacteria, including *Myroides odoratimimus* (Ming et al., 2017), *Sphingobacterium* sp. (Ghosh et al., 2009, 2014), and *Flavobacterium psychrophilum* (Duchaud et al., 2018), and metagenomic samples including Chinese soil (Wang et al., 2017), human feces (Ohashi and Fujisawa, 2017), and hospital wastewater (Wang et al., 2018). The *tetX* gene is encountered in a wide range of ecosystems (human gut, soil, hospital wastewater) and is present on mobile genetic elements primed for horizontal gene transfer. This pattern of ARG dissemination is consistent with horizontal gene transfer of *tetX* between environmental bacteria and human pathogens, as has been observed for many other classes of ARGs (D’Costa et al., 2006; Forsberg et al., 2012; Crofts et al., 2017).

In 2015, a comprehensive functional metagenomic survey using tetracycline selection identified a new family of *tetX* homologs denoted as the tetracycline destructases (*tet49*–*tet55*) (Forsberg et al., 2015). The novel tetracycline destructase genes showed at most 24.4% amino acid sequence homology to TetX. Cloning, heterologous expression, and *in vitro* characterization of Tet49–Tet55 revealed that all nine enzymes were functional tetracycline-inactivating FMOs. Comparative gene analysis revealed a tenth tetracycline destructase gene, *tet56*, in the genome of the human pathogen *Legionella longbeachae*. Antibacterial susceptibility and *in vitro* tetracycline degradation assays proved that *tet56* is a true ARG that confers tetracycline resistance when expressed in *L. longbeachae*. This expanded set of tetracycline destructases provided a unique opportunity to systematically explore substrate selectivity, characterize degradation products, screen for inhibitors, and compare structural features across the enzyme family. These studies led to several important crystal structures of Tet50, Tet51, Tet55, and Tet56 in a variety of functional states (see section “Structural Basis for Tetracycline Inactivation”) that provide mechanistic insight on the diverse oxidation patterns at play for tetracycline substrates (the section “Mechanisms of Tetracycline Oxidation”). These studies also led to the discovery of the first pan tetracycline destructase inhibitor that rescues tetracycline activity when co-administered to tetracycline destructase expressing bacteria (see section “Tetracycline Destructase Inhibitors, an Adjuvant Approach”) (Park et al., 2017).

## Structural Basis for Tetracycline Inactivation

### Anatomy of a Tetracycline Destructase

**FIGURE 4:**
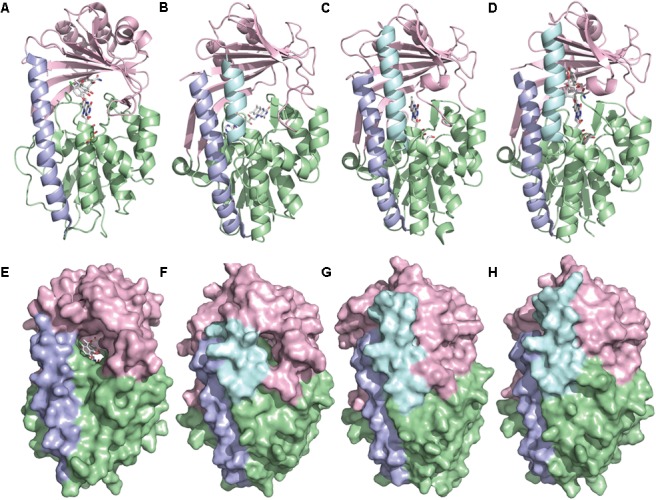
X-ray crystal structure of a tetracycline destructase with bound tetracycline substrate and flavin cofactor. The mobility of the flavin cofactor is highlighted by showing the FAD-IN and FAD-OUT conformations observed during structural studies. **(A)** X-ray crystal structure of CTc bound to TetX (FAD-IN conformation, PDB ID: 2y6r). **(B)** X-ray crystal structure of Tet50 with no bound substrate (FAD-OUT conformation, PDB ID: 5tue). **(C)** X-ray crystal structure of Tet50 with no bound substrate (FAD-IN conformation, PDB ID: 5tue). **(D)** X-ray crystal structure of CTc bound to Tet50 (FAD-IN conformation, PDB ID: 5tui). **(E)** Surface view of X-ray crystal structure of CTc bound to TetX (FAD-IN conformation, PDB ID: 2y6r). **(F)** Surface view of X-ray crystal structure of Tet50 with no bound substrate (FAD-OUT conformation, PDB ID: 5tue). **(G)** Surface view of X-ray crystal structure of Tet50 with no bound substrate (FAD-IN conformation, PDB ID: 5tue). **(H)** Surface view of X-ray crystal structure of CTc bound to Tet50 (FAD-IN conformation, PDB ID: 5tui). Images were generated using PyMOL v1.7.

TetX and all members of the tetracycline destructase family are structural homologs of class A FMOs. Class A FMOs are single component flavoprotein hydroxylases that utilize FAD cofactors and NAD(P)H electron donors to oxidize small molecule substrates—primarily through electrophilic hydroxylation of electron-rich olefins or aromatic rings by a transient, catalytic C4a-hydroperoxyflavin (*vide supra*, **Figure 3**) (van Berkel et al., 2006; Montersino and Berkel, 2013; Huijbers et al., 2014; Mascotti et al., 2016; Romero et al., 2018). In general, this particular type of FMO enzyme is characterized by a single Rossmann fold that binds FAD through non-covalent interactions with the adenosine monophosphate moiety, which is linked to the catalytic isoalloxazine fragment via a polyoxygenated alkyl chain. Flexibility in this alkyl linker is fundamentally important to the success of the catalytic cycle, which involves multiple, dynamic conformational changes in enzyme structure to establish distinct functional enzyme states differentiated by relative FAD activation and three-dimensional orientation (*vide infra*). TetX and members of the tetracycline destructase family are structurally similar and functionally homologous (Forsberg et al., 2015). As shown in **Figure 4**, the tetracycline-inactivating FMO enzymes are composed of two major domains – a lower FAD-binding domain (green) that exhibits the characteristic Rossmann fold and an upper tetracycline binding domain (pink) (Volkers et al., 2011; Park et al., 2017). The association of the two domains is stabilized by a C-terminal alpha-helix (purple), and specifically in the case of the tetracycline destructase family, a second C-terminal alpha-helix (cyan) is present near the tetracycline binding site, which plays an important role in substrate recognition and loading (Park et al., 2017).

While the exact sequence of events involving dynamic conformational changes to the enzyme during the catalytic cycle are currently unknown (see section “Tetracycline Destructase Inhibitors, an Adjuvant Approach” for a proposed model), two enzyme conformers have been observed via X-ray crystallographic analysis which are distinct in both FAD-orientation and tertiary protein structure (**Figure 4**). The FAD-OUT conformer, in which the substrate loading channel is open and the FAD cofactor is pointed away from the tetracycline binding domain, allows for easy accommodation of the substrate and ready access of FAD to electron-donor NADPH to maintain a steady concentration of reduced FADH_2_ primed for reactivation with molecular oxygen (shown for Tet50, **Figure 4B**, surface view **Figure 4F**). While the FAD-OUT conformation has not been experimentally observed for TetX, it has been observed in other class A-type FMO-enzymes (particularly StaC and RebC) (Ryan et al., 2008; Goldman et al., 2012) and is fundamentally important to maintain catalytic efficiency and relevant levels of antibiotic resistance.

Upon substrate and/or NADPH accommodation, several class A FMO enzymes undergo a series of discrete conformational changes that flip the activated FADH_2_ toward the bound substrate and allow for both the protected formation of the reactive C4a-peroxyflavin from FADH_2_ and molecular oxygen and subsequent substrate oxidation (Ghisla and Massey, 1989; Palfey and McDonald, 2010; Montersino and Berkel, 2013; Huijbers et al., 2014). However, this FAD-IN conformer has been observed via X-ray crystallography for TetX *and* Tet50 in the *absence* of NADPH and substrate. A defined sequence of mechanistic events has been elucidated for prototypical class A FMO *p*-hydroxybenzoate hydroxylase (Eppink et al., 1998, 1999; Suemori, 2013). While the tetracycline-inactivating enzymes appear to be class A FMOs, the defined sequence of events, including NADPH-binding elements, and relevant extrapolation of these no-substrate, FAD-IN conformers to solution-phase enzyme dynamic processes remain currently unknown. Nevertheless, X-ray crystallographic analysis of the no substrate- and substrate-bound FAD-IN conformers of Tet50 and the substrate-bound FAD-IN conformer of TetX highlights several structural differences that may aid in the explanation of the unique, enzyme-specific antibiotic resistance phenotypes observed *in vitro* and in whole cell for each of these tetracycline-inactivating enzymes (Forsberg et al., 2015; Park et al., 2017).

In the absence of the second C-terminal “gatekeeper” helix observed in members of the tetracycline destructase families, the FAD-IN conformation for CTc bound to TetX utilizes several hydrophobic, mostly aromatic residues to shield the FAD-complex from C4a-peroxyflavin-reactive solvent molecules (**Figure 4A**; Volkers et al., 2011). Indeed, the FAD cofactor is barely visible in the surface view of the CTc-bound, FAD-IN conformer of TetX (**Figure 4E**). However, a small, open pocket near the substrate-binding site allows for a portion of the substrate – in this case, CTc – to extend from the active site of the enzyme into solvent exposed space. In contrast, the substrate loading channel closes completely in the no substrate- and CTc-bound FAD-IN conformers of Tet50, where both the FAD and the substrate are shielded from solvent interaction by both the “gatekeeper helix” and a hydrophobic phenylalanine residue in the substrate-binding domain (Phe95, **Figures 4C,D**) (Park et al., 2017). This structural difference between FAD-IN conformers of TetX and Tet50 is highlighted in the surface views of each protein conformer shown in **Figure 4** (TetX **Figure 4E** and Tet50 **Figures 4G,H**). In addition, the structure variability in FAD-IN conformation has important implications in substrate recognition and binding, as well as enzyme–substrate specificity and preference, that directly result in distinct tetracycline degradation profiles.

### Diverse Substrate-Binding Modes

As is the case with most class A FMO enzymes (van Berkel et al., 2006; Montersino and Berkel, 2013; Huijbers et al., 2014; Romero et al., 2018), the position of substrate oxidation is heavily dependent on the spatial orientation of the bound substrate in relation to the transient enzyme-associated C4a-peroxyflavin cofactor. Because active site flexibility can lead to product mixtures (as multiple binding modes can lead to multiple degradation products), it is important to correlate experimentally observed binding modes with potential sites of substrate oxidation that correspond to characterized oxidation products.

**FIGURE 5:**
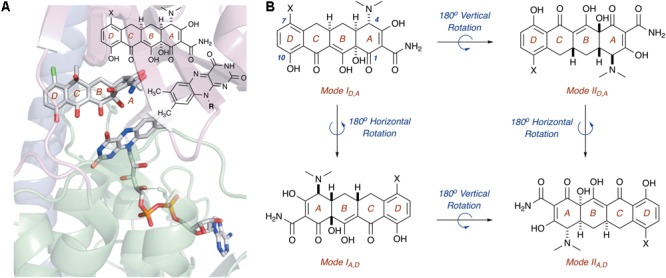
**(A)** X-ray crystal structure of CTc bound to TetX in binding mode I_D,A_ defines the orientation of FAD relative to each CTc binding mode (PDB ID: 2y6r). **(B)** Theoretical and experimentally observed tetracycline binding modes (four total). Image in panel **(A)** was generated using PyMOL v1.7.

In this regard, the X-ray crystal structure of CTc bound to TetX can serve as a point of reference to help define spatial coordinates within the enzyme active site in which the tetracycline substrate can rotate/bind (**Figure 5**; Volkers et al., 2011). As is shown in **Figure 5A**, enzyme-bound CTc is located above the FAD cofactor, which is extended toward the substrate-binding domain within the enzyme active site, as is consistent with the FAD-IN conformation. In addition, CTc is oriented in such a way that the A-ring (C1 proximal, C4 distal) is closest to the FAD cofactor, while the D-ring lies nearer the C-terminal alpha-helix (C10 proximal, C7 distal). Thus, this orientation can be defined as mode I_D,A_, where the mode number (I or II) describes the proximal or distal position of the C1–C10 hemisphere of the molecule in relation to the FAD cofactor, and the subscript identifier describes the west-to-east (left-to-right) association of the D- and A-rings of the tetracycline substrate in relation to the FAD cofactor. Correspondingly, a 180° horizontal rotation of the tetracycline substrate bound in mode I_D,A_ (about a vertical axis) would result in substrate-binding mode I_A,D_ (C1,D-ring proximal), where the sole modification in binding mode is the relation of the A-ring (now west) and D-ring (now east) to the FAD cofactor (**Figure 5B**). The association of the C1–C10 hemisphere to the FAD isoalloxazine remains unchanged. In contrast, a 180° vertical rotation (about a horizontal axis) of the tetracycline substrate bound in mode I_D,A_ would result in the substrate-binding mode II_D,A_, where the sole modification in binding mode is the relation of the C1–C10 hemisphere of the molecule in relation to the FAD cofactor (now distal). The association of the A- and D-rings to the FAD isoalloxazine remains unchanged. In this way, four potential binding modes for tetracycline substrates – modes I_D,A_, I_A,D_, II_D,A_, and II_A,D_ – can be defined, and the oxidation of the tetracycline substrate will be binding-mode specific.

**FIGURE 6:**
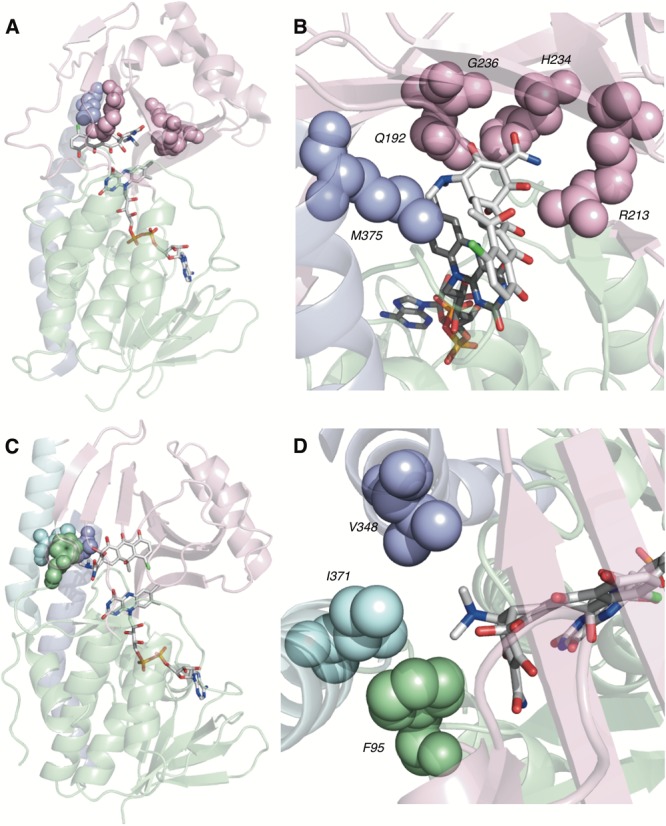
Recognition elements of CTc A-ring for each experimentally observed substrate-binding mode. **(A)** X-ray structure of CTc bound to TetX in Mode I_D,A_ (PDB ID: 2y6r). **(B)** Expanded X-ray structure of CTc bound to TetX in Mode I_D,A_ with interacting structural residues highlighted and labeled (PDB ID: 2y6r). **(C)** X-ray structure of CTc bound to Tet50 in Mode II_A,D_ (PDB ID: 5tui). **(D)** Expanded X-ray structure of CTc bound to Tet50 in Mode II_A,D_ with interacting structural residues highlighted and labeled (PDB ID: 5tui). Images were generated using PyMOL v1.7.

Of the four potential substrate-binding modes, only two have been experimentally observed via X-ray crystallography for the binding of substrates to tetracycline-inactivating enzymes (**Figure 6**; Volkers et al., 2011; Park et al., 2017). As described in the previous paragraph, CTc binds to TetX in mode I_D,A_, and the primary substrate recognition elements are located in the substrate-binding domain, where hydrogen-bond donating residues (Q192, H234, and R213) interact with H-bond accepting ketone and amide functional groups on the A-ring of CTc (**Figures 6A,B**). While a number of hydrophobic residues in the substrate-binding domain also interact with the C- and D-rings of the enzyme-bound CTc (Volkers et al., 2011), the open cavity near the substrate loading channel of the FAD-IN conformer of TetX allows mostly weak interactions with the D-ring – which can also associate with readily available solvent molecules. In contrast, as shown in **Figure 6C**, CTc binds to Tet50 in mode II_A,D_ (FAD-IN conformer shown), where several van der Waals interactions between the C-terminal stabilizing alpha-helix, the second C-terminal “gatekeeper” helix, and a residue of the lower FAD-binding domain interact with the *N,N′*-(dimethyl)amino substituent of the now “west” tetracycline A-ring. The expanded view of the mode II_A,D_ in **Figure 6D** highlights the important contribution of the second C-terminal “gatekeeper” helix – which is present in members of the tetracycline destructase family of enzymes but noticeably absent in TetX – to substrate recognition and accommodation. In turn, the substantial differences in experimentally observed substrate-binding modes for CTc-bound TetX and Tet50 account for the variability in tetracycline degradation profiles observed for both enzymes, as the proximities of enzyme-reactive functional groups to the C4a-center of the FAD cofactor directly influence the nature of potential degradation cascades (Forsberg et al., 2015; Park et al., 2017).

## Mechanisms of Tetracycline Oxidation

### Oxidative “Soft” Spots on the Tetracycline Scaffold

Due to the unstable nature of tetracyclines to light (Moore et al., 1983; Halling-Sorensen et al., 2002; Fuoco, 2015), heat (Nguyen et al., 2015), and acid or base (Yuen and Sokoloski, 1977), the enzymatic and non-enzymatic degradation profiles of tetracycline antibiotics are complex. The mixtures of products resulting from tetracycline oxidation are likely responsible for the distinct brown colored growth phenotype of *E. coli* expressing tetracycline destructases (Speer and Salyers, 1989; Yang et al., 2004; Forsberg et al., 2015). However, because the spatial orientation of the substrate in relation to the reactive C4a-peroxyflavin is fundamentally important to the mechanism of oxidation and tetracycline degradation, the experimentally observed binding modes of CTc bound to TetX and Tet50 can be used to identify potential, binding mode-specific, oxidative “soft” spots on the tetracycline scaffold. These oxidative “soft” spots can then be used as starting points to propose potential non-enzymatic degradation cascades to explain experimentally observed degradation profiles (Yang et al., 2004; Forsberg et al., 2015; Park et al., 2017).

**FIGURE 7:**
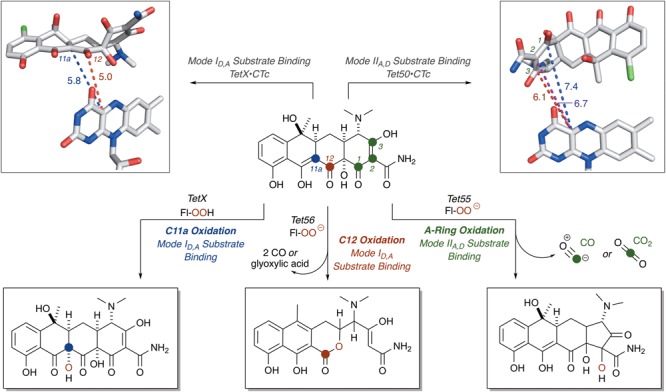
Victim of fate: the site of tetracycline oxidation is determined by binding mode and distance from flavin-C4a. Bond distances to reactive centers on CTc bound to TetX in Mode I_D,A_ (PDB ID: 2y6r) and CTc bound to Tet50 in Mode II_A,D_ (PDB ID: 5tui) were determined in PyMOL from the corresponding PDB files. Images of FAD were generated using PyMOL v1.7.

For CTc bound in mode I_D,A_, the proposed potential oxidative sites on CTc are the C11a-enol- and C12-carbonyl-carbon centers, at distances of 5.8 and 5.0 Å, respectively, from the C4a-position on the FAD cofactor (as determined from the X-ray crystal structure of CTc bound to TetX, **Figure 7**). This is consistent with the enzymatic hydroxylation of the C11a-center of oxytetracycline by TetX reported by Wright and coworkers in 2004, where acid-stabilizing hemiketal formation of the enzymatic degradation product allowed the authors to isolate and fully characterize the intermediate (see **Figure 3**, *vide supra*). For CTc bound in mode II_A,D_, where the A-ring is most accessible to C4a-peroxyflavin oxidation, the proposed potential oxidative sites on CTc are the C1-carbonyl, C2-enol, and C3-carbon centers at distances of 7.4, 6.7, and 6.1 Å, respectively (as determined from the X-ray crystal structure of CTc bound to Tet50, **Figure 7**). It appears that the tetracycline substrate is merely a victim of fate and the oxidative “soft spot” that aligns closest to the C4a-peroxyflavin will be oxidized. Properly defining the distance constraints between flavin-C4a and oxidation sites will enable some predictive capacity. A similar oxidative “soft spot” has been reported for the rifamycin monooxygenase (Rox) that hydroxylates the C2 position of the hydroxynaphthol leading to formation of a 1,2-naphthoquinone (Koteva et al., 2018; Liu et al., 2018). In the rifamycin-Rox structure C2 is reported to be 4.7 Å from flavin-C4a.

### Oxidation Initiates a Cascade of Chemistry

The highly conjugated nature of the tetracycline antibiotics enables chemical communication across the entire 4-ring structure, which – in turn – can result in a variety of non-enzyme catalyzed rearrangement cascades following the enzymatic oxidation of tetracycline substrates. Indeed, the complex nature of the enzymatic degradation profiles of tetracycline antibiotics and instability of oxidized degradation products implies that non-enzymatic cascade reactions must occur spontaneously in solution to result in a decrease of observed enzymatic degradation product. While the primary enzymatic degradation product of TetX monohydroxylation of oxytetracycline has been observed (Yang et al., 2004), several degradation cascades have been proposed to explain LCMS-observed degradation product formation resulting from the tetracycline destructase-mediated enzymatic oxidation of binding-mode-specific reactive “soft spots” on tetracycline scaffolds (Forsberg et al., 2015; Park et al., 2017).

**FIGURE 8:**
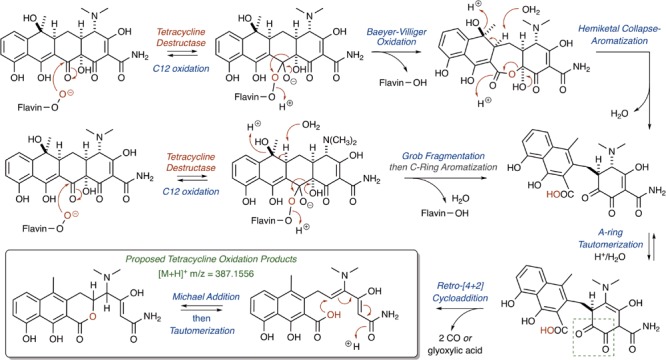
Cascade reactions leading to tetracycline degradation products from enzymatic C12-oxidation of *mode I_D,A_*-bound tetracycline.

The nucleophilic or electrophilic nature of the intermediate C4a-peroxyflavin (pKa ∼ 7–8; Favaudon, 1977; Kemal et al., 1977) within the enzyme active site can be ambiguous across class A FMO enzymes and is largely dependent upon protonation state (protonated C4a-peroxyflavin = electrophilic species; deprotonated C4a-peroxyflavin = nucleophilic species; Massey, 1994; Montersino and Berkel, 2013; Huijbers et al., 2014). The majority act as electrophiles in the hydroxylation of electron-rich aromatic rings (Wierenga et al., 1979), but Baeyer–Villiger chemistry has been observed when the substrate is an electrophilic carbonyl (Ryerson et al., 1982; Schwab et al., 1983; Walsh and Wencewicz, 2013). Thus, for the Tet56-mediated degradation of tetracycline resulting in the formation of major ion [M+H]^+^ 387.1556, it is proposed that a nucleophilic C4a-flavinperoxide can add to the C12-ketone of tetracycline to form a transient, tetrahedral intermediate (**Figure 8**; Forsberg et al., 2015). This intermediate can undergo a Baeyer–Villiger-type ring expansion via a 1,2-alkyl-shift to eliminate the C4a-hydroxyflavin and provide an intermediate ester, which upon hemiketal collapse and rearomatization of the former C-ring can provide a naphthyl-substituted cyclohex-4-en-1,2-dione intermediate. Alternatively, the same tetrahedral intermediate can undergo a Grob fragmentation, followed by C-ring aromatization, to arrive at the same naphthyl-substituted cyclohex-4-en-1,2-dione intermediate. This cyclohex-4-en-1,2-dione can then tautomerize and undergo a retro[4+2]-cycloaddition to eliminate either two equivalents of carbon monoxide (CO) or one equivalent of transient oxoketene – that, upon hydrolysis, would provide an equivalent of glyoxylic acid – to afford the proposed degradation product as the naphthylic acid ([M+H]+ *m*/*z* = 387.1556). Upon Michael addition and enol tautomerization, the naphthylic acid intermediate can provide the corresponding lactone ([M+H]+ *m*/*z* = 387.1556).

**FIGURE 9:**
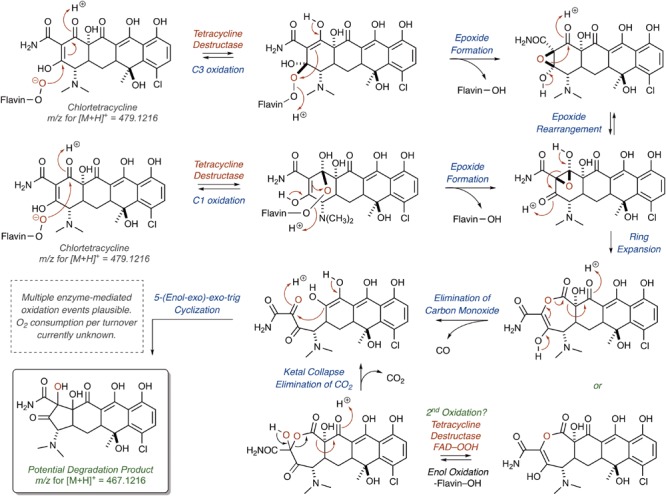
Cascade reactions leading to tetracycline degradation products from enzymatic C1- or C3-oxidation of *mode II_A,D_*-bound tetracycline.

Correspondingly, for the Tet55-mediated degradation of tetracycline resulting in the formation of major ion [M+H]^+^ 467.1216, it is proposed that the nucleophilic addition of the C4a-flavinperoxide to either the C1- or C3-positions of the tetracycline substrate can result in the formation of two, rapidly interconverting epoxide species (**Figure 9**;Park et al., 2017). These species can undergo a ring-expansion via collapse of the hemiketal-epoxide to provide an intermediate lactone, which upon elimination of one equivalent of CO and subsequent intramolecular 5-(enol-exo)-exo-trig cyclization of the resultant enol-containing alpha-ketoamide, could be converted to the proposed degradation product ([M+H]^+^ 467.1216). Alternatively, the intermediate lactone could undergo a second enol oxidation, followed by ketal collapse and extrusion of carbon dioxide (CO_2_), to provide the same enol-containing alpha-ketoamide, which after intramolecular 5-(enol-exo)-exo-trig cyclization provides the corresponding degradation product.

**FIGURE 10:**
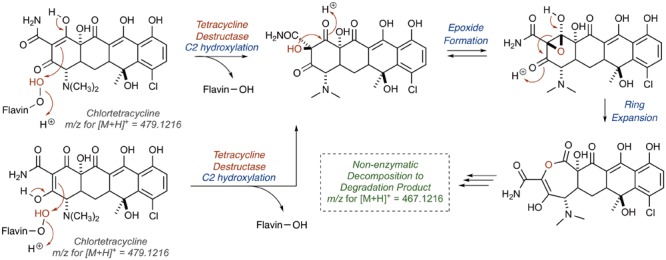
Alternative mechanistic pathway leading to formation of the ring contracted degradation product ([M+H]^+^ 467.1216) initiated by hydroxylation of C2.

Alternatively, hydroxylation of C2 with an electrophilic C4a-peroxyflavin would initiate a cascade resulting in the same degradation product (**Figure 10**; [M+H]^+^ 467.1216). A similar hydroxylation of C2 in mithramycin biosynthesis initiates a ring opening cascade to provide the bioactive form of the DNA minor groove-binding molecule (Gibson et al., 2005). While the precise degradation products remain unknown for both the enzymatic oxidation and the following non-enzymatic degradation cascade, these mechanistic proposals may serve as useful models as more information becomes available en route to the elucidation of the enzymatic degradation of tetracycline antibiotics (Yang et al., 2004; Forsberg et al., 2015; Ghosh et al., 2015; Park et al., 2017). It is noteworthy that a similar cascade event takes place for the Rox-mediated inactivation of rifamycin where oxidation of the C2 position of the hydroxynaphthalene leads to ring opening of the macrolactam and subsequent linearization of rifamycin (Koteva et al., 2018; Liu et al., 2018). A detailed understanding of enzymatic and non-enzymatic degradation cascades for tetracycline and other antibiotics is critical for designing future generations of molecules that overcome these resistance mechanisms and diagnostic tools to detect active antibiotic-inactivating enzymes in clinical samples. In fact, the degradation mechanisms of beta-lactam antibiotics by beta-lactamase enzymes were fundamentally important to the design of fluorogenic and chromogenic probes used in clinical diagnostic applications (O’Callaghan et al., 1972; Yu et al., 2012; Ghavami et al., 2015).

## Tetracycline Destructase Inhibitors, an Adjuvant Approach

### Antibiotic Destructase Inhibitors

There are two clinically proven approaches to overcoming resistance by antibiotic destructases: (1) modification of the antibiotic structure in a manner that prevents covalent modification (i.e., successive generations of beta-lactams) (Fisher et al., 2005); (2) co-administration of an adjuvant that inhibits production and/or catalytic activity of antibiotic destructases (i.e., beta-lactam/beta-lactamase inhibitor combinations) (Bush, 2018). Modern beta-lactam antibiotics are now fifth generation scaffold iterations, and it is rare to push new beta-lactams into the clinic without co-administration of a beta-lactamase inhibitor. The first beta-lactamase inhibitors, such as clavulanic acid isolated from *Streptomyces clavuligerus*, were found to be beta-lactams like the parent antibiotic (Reading and Cole, 1977). Nature seems to have invented this adjuvant approach long before medicinal chemists ever proposed the idea. In addition to clavulanic acid, *S. clavuligerus* also produces the cephalosporin antibiotic cephamycin. The biosynthetic genes for both clavulanic acid and cephamycin are colocalized in a “super cluster” operon, resulting in simultaneous production of the antibiotic and adjuvant to ensure efficacy against beta-lactamase-producing competitors (Ward and Hodgson, 1993). It is conceivable that tetracycline producers can also biosynthesize tetracycline destructase inhibitors to protect the tetracycline antibiotic, though evidence of which has yet to be discovered.

### Anhydrotetracycline – The First Tetracycline Destructase Inhibitor

**FIGURE 11:**
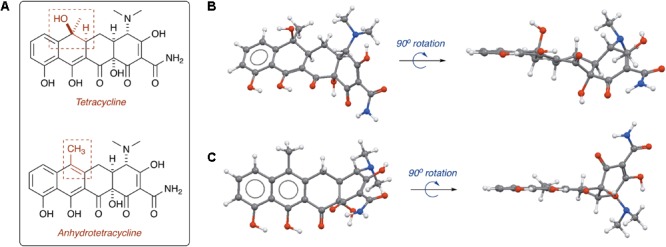
**(A)** Structures of tetracycline (top) and anhydrotetracycline (bottom). Conformation of tetracycline **(B)** and anhydrotetracycline **(C)** as viewed from face and edge of the tetracyclic core. 3D structures of tetracycline and anhydrotetracycline in panels **(B)** and **(C)** were energy minimized using Spartan and images were generated using Mercury software v3.10.

Tetracycline producers readily excrete analogs and shunt products during tetracycline biosynthesis (Pickens and Tang, 2010; Wang et al., 2013). One intermediate and shunt product in tetracycline biosynthesis, anhydrotetracycline, was found to be a poor substrate for the tetracycline destructases (Forsberg et al., 2015; Park et al., 2017). Only TetX was able to oxidize anhydrotetracycline, albeit very slowly, suggesting that tetracycline destructases can still bind anhydrotetracycline in the substrate-binding domain despite the subtle structural differences compared to the parent tetracycline (**Figure 11**). Dehydration of the tetracycline scaffold at C6 provides the more hydrophobic anhydrotetracycline with a flattened naphthalene C,D-ring system and some conformational changes in the A,B-rings. Despite the subtle structural differences, tetracycline and anhydrotetracycline show remarkably different biological activity. Tetracyclines are potent ribosome inhibitors and have an overall bacteriostatic effect on cells (Wilson, 2009). Anhydrotetracyclines are weak ribosome inhibitors and have a bactericidal effect on cells, presumably through membrane depolarization (Rasmussen et al., 1991; Oliva et al., 1992). Anhydrotetracycline was able to rescue the activity of tetracyclines when co-administered in checkerboard antibacterial assays against *E. coli* expressing tetracycline destructases (Park et al., 2017). Furthermore, anhydrotetracycline was shown to be a potent inhibitor of tetracycline destructases *in vitro* at low micromolar levels. It remains unclear if anhydrotetracycline is acting as a true competitive inhibitor or a competitive (slow) substrate. These initial studies suggest that anhydrotetracycline is a viable tetracycline destructase lead inhibitor and sets the stage for developing tetracycline destructase inhibitors as adjuvants for use in combination therapy with tetracycline antibiotics. This also raises the question as to whether tetracycline-producing microbes excrete anhydrotetracycline with the tetracycline antibiotic to act synergistically as tetracycline destructase inhibitors and secondary antibiotics with an alternate mode of action (membrane depolarization). Mixtures of tetracycline and tetracycline degradation products, including anhydrotetracycline, have been shown to invert resistance selection and select against tetracycline efflux pumps (Palmer et al., 2010; Chait et al., 2011; Stone et al., 2016). Tetracycline destructases and associated degradation products might play a variety of roles beyond resistance in natural environments, including signaling and control of microbial populations (Yim et al., 2007).

### Structural and Mechanistic Basis for Inhibition

**FIGURE 12:**
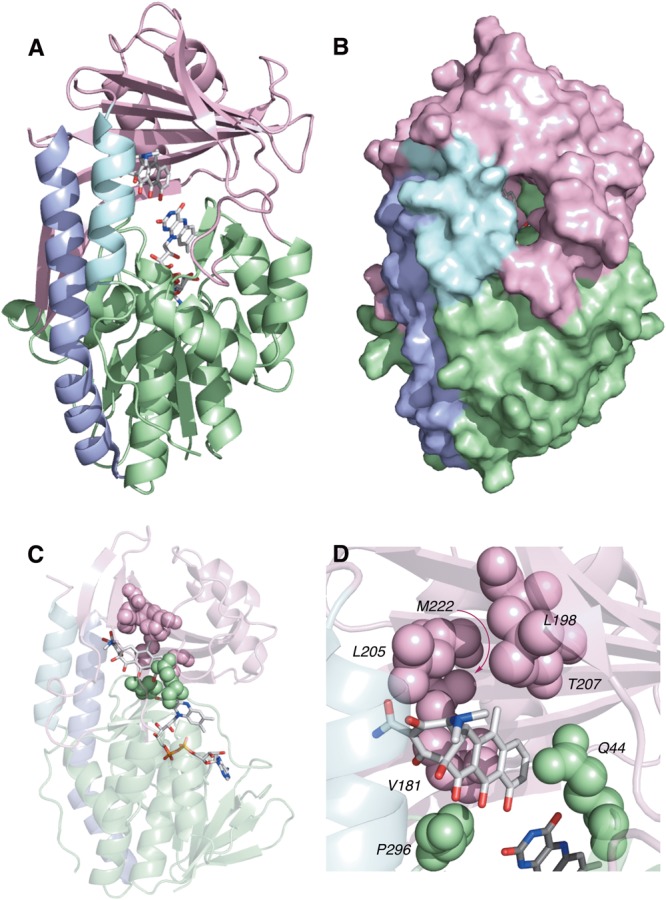
**(A)** X-ray crystal structure of anhydrotetracycline bound to Tet50 in Mode I_A,D_ (PDB accession number 5tuf). **(B)** Surface view of X-ray crystal structure of aTC bound to Tet50. **(C)** X-ray crystal structure of anhydrotetracycline bound to Tet50 in Mode I_A,D_ with recognition residues highlighted. **(D)** Expanded X-ray crystal structure of anhydrotetracycline bound to Tet50 in Mode I_A,D_ with recognition residues highlighted and labeled. Images were generated using PyMOL v1.7.

X-ray crystal structures of anhydrotetracycline bound to Tet50 revealed several important distinctions in the binding mode compared to the previously discussed structures of CTc bound to Tet50 and TetX (**Figure 12**; Park et al., 2017). First, a new binding orientation of the tetracyclic scaffold, Mode I_A,D_, was observed (see **Figure 5** for reference). The flattened C,D-ring system enables anhydrotetracycline to bind deeper in the substrate-binding domain with the C6-methyl group filling a hydrophobic pocket lined by L198, T207, L205, M222, V181, P296, and Q44. This orientation pushes the flavin “out” and orients the gatekeeper helix so the active site is open to solvent. This binding mode is inaccessible to canonical tetracyclines with methylation and hydroxylation at C6 due to steric clashing. The anhydrotetracycline-stabilized Tet50 conformation is predicted to be catalytically incompetent; however, other binding modes with anhydrotetracycline might be possible based on the observed plasticity of the tetracycline destructases for CTc. Since TetX can slowly oxidize anhydrotetracycline, it seems possible that anhydrotetracyclines can bind in alternate modes similar to CTc that might enable the flavin cofactor to reach the catalytically competent “in” conformation. The gatekeeper helix might be the distinguishing structural feature between TetX and other tetracycline destructases that determines conformational dynamics, substrate plasticity, catalytic efficiency, and susceptibility to inhibition. The structure of anhydrotetracycline bound to Tet50 should serve as a guide for structure-based drug design of improved tetracycline destructase inhibitors.

### Mechanistic Model for Catalysis and Inhibition

**FIGURE 13:**
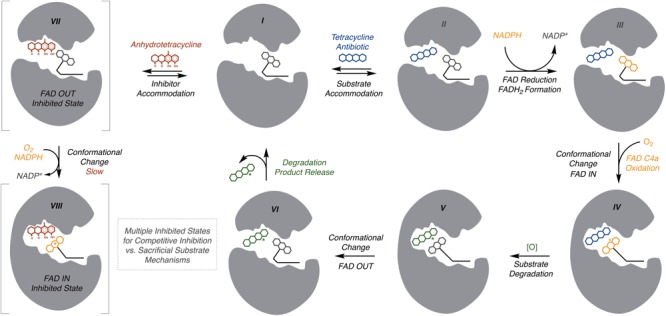
A mechanistic model for the tetracycline destructase catalytic cycle and inhibition by anhydrotetracycline is proposed. (I) Flavin oxidized, open active site; (II) substrate binding, flavin oxidized, open active site; (III) substrate bound, flavin reduced, open active site; (IV) substrate bound with C4a-peroxyflavin in “in” conformation, closed active site; (V) oxidized product bound with C4a-hydroxyflavin in “in” conformation, closed active site; (VI) substrate bound with C4a-hydroxyflavin in “out” conformation, open active site; (VII) inhibitor bound with flavin in “out” conformation, open active site; (VIII) inhibitor bound with C4a-peroxyflavin in “in” conformation, closed active site.

Based on the structural (the section “Structural Basis for Tetracycline Inactivation”), mechanistic (the section “Mechanisms of Tetracycline Oxidation”), and inhibition (the section “Tetracycline Destructase Inhibitors, an Adjuvant Approach”) studies of the tetracycline destructases, a generic model for the catalytic cycle of tetracycline inactivation and inhibition is proposed (**Figure 13**). The tetracycline destructase can exist in a resting state with the flavin in the oxidized form (I). As shown for other class A FMOs (Abdelwahab et al., 2016), substrate binding (II) can accelerate flavin reduction by NADPH (III) with the flavin dynamically moving between the FAD-IN and FAD-OUT states. The timing and location of C4a-peroxyflavin formation is unclear, but presumably, to oxidize the substrate, the C4a-peroxyflavin must move to the “in” conformation (IV). If the tetracycline destructase has a gatekeeper helix, then the enzyme active site will be closed when the flavin transitions from “out” to “in” and ultimately is positioned to oxidize the tetracycline substrate (V). Movement of the flavin to the “out” conformation will result in movement of the gatekeeper helix to open the active site and release the tetracycline product to complete the catalytic cycle (VI). The oxidized tetracycline products might be subject to further enzymatic oxidation or non-enzymatic cascade reactions leading to non-antibacterial tetracycline degradation products. Anhydrotetracycline is predicted to competitively bind in the substrate-binding domain, which can lead to formation of a stabilized tetracycline destructase inhibition complex with the flavin cofactor essentially “locked” in the unproductive “out” conformation (VII). Anhydrotetracycline is slowly oxidized by TetX; thus, it is conceivable that net destructase inhibition is achieved by anhydrotetracycline acting as a competitive (slow) substrate (VIII). FMOs are a diverse family of oxidoreductases that perform a staggering array of transformations (Walsh and Wencewicz, 2013). There are still many unanswered questions regarding the timing and mechanism of tetracycline inactivation and tetracycline destructase inhibition that will require further structural, mechanistic, and kinetic studies (Eswaramoorthy et al., 2006; van Berkel et al., 2006; Romero et al., 2018).

## Final Thoughts

### Tetracycline Destructases Represent an Emerging Threat to Next-Generation Tetracyclines

Antibiotic resistance is a moving target (Wright, 2007). Tetracyclines have kept pace through advancements in scaffold derivatization, semi-synthesis, biosynthesis, and total chemical synthesis (Chopra and Roberts, 2001; Liu and Myers, 2016; Sun and Xiao, 2017). Increased use of third (tigecycline) and fourth generation (eravacycline, omadacycline) tetracyclines that overcome resistance by efflux and ribosome protection threaten to select for new resistance mechanisms. The tetracycline destructases are FMOs that confer resistance to these next-generation tetracyclines via covalent inactivation (Moore et al., 2005; Grossman et al., 2012; Sutcliffe et al., 2013; Volkers et al., 2013). Antibiotic oxidation is an emerging inactivation resistance strategy that has only been observed for one other antibiotic class, the rifamycins (Abdelwahab et al., 2016; Liu et al., 2016, 2018; Koteva et al., 2018). Resistance to rifamycin via enzymatic inactivation is not limited to FMOs; in fact, known rifamycin destructases include FMOs (Abdelwahab et al., 2016; Liu et al., 2016, 2018; Koteva et al., 2018), glycosyltransferases (Spanogiannopoulos et al., 2012), ADP-ribosyltransferases (Baysarowich et al., 2008), and phosphotransferases (Stogios et al., 2016). Future prospecting for tetracycline ARGs will likely result in the discovery of non-FMO tetracycline destructases. Tetracyclines, and rifamycins, are sensitive to chemical photooxidation; so, it seems appropriate that the first tetracycline destructases, FMOs, exploit this reactivity (Moore et al., 1983). The relevance of FMO tetracycline destructases is presumably limited to aerobic infections due to the strict requirement of molecular oxygen for tetracycline inactivation (Guiney et al., 1984). Historically, tetracyclines have been found to be more effective against aerobic bacteria than anaerobic bacteria (Chow et al., 1975). Thus, acquisition and expression of tetracycline destructase FMO genes will be beneficial for aerobic and facultatively anaerobic pathogens that cause a variety of aerobic infections, including pulmonary, periodontal, skin, and post-surgical wound infections (Chopra and Roberts, 2001).

### Functional Prospecting Is Needed to Monitor the Dissemination of Tetracycline Destructase Genes in Human Pathogens

**FIGURE 14:**
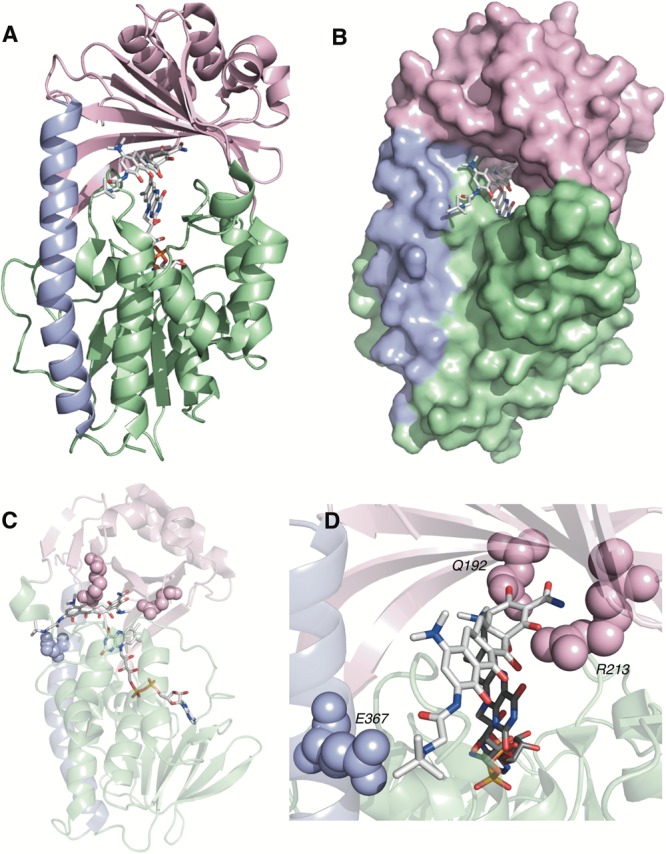
**(A)** X-ray structure of tigecycline bound to TetX in Mode I_D,A_ (PDB accession number 4a6n). **(B)** Surface view of X-ray structure of tigecycline bound to TetX in Mode I_D,A_. **(C)** X-ray structure of tigecycline bound to TetX in Mode I_D,A_ with relevant substrate recognition interactions highlighted. **(D)** Expanded X-ray structure of tigecycline bound to TetX in Mode I_D,A_ with relevant substrate recognition interactions highlighted for the A-ring (Q192, R213) and the D-ring *N*-*t*-butyl-glycylamide substituent (E367). Electron density for the C2-carboxamide bond was missing in the PDB file 4a6n. The C2-carboxamide bond was added using the create bond function in PyMOL. Images were generated using PyMOL v1.7.

Tetracycline destructases have emerged on mobile genetic elements in human bacterial pathogens (Leski et al., 2013). It appears urgent to have a management plan for tetracycline destructases in place before a clinical crisis emerges. Functional metagenomics is an effective strategy to monitor the dissemination of tetracycline destructases in hospitals and should be continuously applied to patient samples and clinical isolates (Crofts et al., 2017). Tetracycline destructases, including TetX, evolved in the presence of countless tetracycline variants in diverse environments and thus gained great substrate plasticity (Forsberg et al., 2015; Park et al., 2017). This explains the ability of TetX to oxidize never-before-seen synthetic tetracyclines, including the clinical antibiotics tigecycline and eravacycline, through flexibility in substrate-binding mode that allows for accommodation of tetracyclines with bulky D-ring substituents (**Figure 14**; Grossman et al., 2012; Sutcliffe et al., 2013). This same type of substrate plasticity has been well documented for the beta-lactamases and carries the risk of causing pan-resistance to an entire drug class (Bush and Jacoby, 2010). A recent study showed that random mutagenesis of the *tetX* gene readily provided TetX variants with significantly improved activity toward tigecycline inactivation (Linkevicius et al., 2016). This suggests that *tetX* is poised to emerge as a primary resistance mechanism under tigecycline selective pressure. Similar to tigecycline, fourth generation molecules like eravacycline and omadacycline possess bulky D-ring substituents that are accommodated and solvent exposed by the constitutively open TetX active site (**Figure 14**). Comprehensive study of the functional evolution and evolvability of the tetracycline destructases is merited to anticipate future enzyme variants that might emerge in human pathogens (Brandt et al., 2017; Crofts et al., 2017; Pawlowski et al., 2018). It will be interesting to look to tetracycline biosynthetic pathways for FMOs that introduce scaffold oxidations to see if these can undergo gain-of-function as destructases, which might point toward a pathway of evolution for the tetracycline destructases (Gibson et al., 2005; Pickens and Tang, 2010; Walsh and Wencewicz, 2013; Wang et al., 2013).

### Inhibitors Are Needed as Tetracycline Adjuvants for Future Combination Therapy

Mechanistic and kinetic evaluation of tetracycline destructases have revealed an impressive capacity for substrate oxidation at diverse scaffold positions (**Figure 7**). Further studies will be needed to map oxidative soft spots to guide the synthesis of next-generation tetracyclines that block oxidation by tetracycline destructases and maintain high affinity for the bacterial ribosome. The high degree of substrate plasticity of the tetracycline destructases suggests that inhibitors will likely be needed as adjuvants for combination therapies with tetracycline antibiotics. The history of beta-lactamases tells us that scaffold iteration is not enough, and it would be prudent to have inhibitors in hand before tetracycline destructases become a widespread clinical resistance mechanism. Anhydrotetracycline has emerged as the first tetracycline destructase inhibitor and shows potential to be a pan-destructase inhibitor. TetX can slowly oxidize anhydrotetracycline; thus, models as both a competitive inhibitor and a competitive sacrificial substrate should be applied. Most beta-lactamase inhibitors are mechanism-based and act as both sacrificial substrates and covalent inhibitors, providing clinical evidence that this model of destructase inhibition is viable. The crystal structure of anhydrotetracycline bound to Tet50 in a novel binding mode that presumably locks the flavin cofactor in the “out” conformation is a good starting point for structure-based drug design (**Figure 12**; Park et al., 2017). Anhydrotetracyclines do have antibacterial activity as membrane disruptors and are capable of cell permeation even at sub-MIC levels relevant for tetracycline destructase inhibition when used in combination with tetracycline antibiotics (Rasmussen et al., 1991). In addition to anhydrotetracycline, a variety of inhibitor scaffolds would be beneficial, as history from beta-lactamases tells us that multiple inhibitor types will be needed to keep pace with the constantly evolving destructases (Drawz and Bonomo, 2010; Bush, 2018). For antibiotic resistance it is not a question of if, but when it will become a clinical problem, which begs the question: When will we take notice? Given the historical precedence for enzymatic antibiotic inactivation mechanisms to dominate resistance landscapes, it is conceivable that all next-generation tetracyclines will need to be co-administered with a tetracycline destructase inhibitor potentially in our lifetime. Therefore, a proactive approach to developing next-generation tetracyclines and tetracycline destructase inhibitors is the prudent solution to avoiding a clinical crisis … for now.

## Author Contributions

JM and TW wrote the manuscript and prepared the figures.

## Conflict of Interest Statement

The authors have filed a U.S. patent application (application 20170369864) on methods for treating bacterial infections caused by pathogens expressing tetracycline-inactivating enzymes.

## Abbreviations

ARG: antibiotic resistance gene
CTc: chlortetracycline
FAD: flavin adenine dinucleotide
FMO: flavin monooxygenase
NADPH: nicotinamide adenine dinucleotide phosphate reduced form
NADP^+^: nicotinamide adenine dinucleotide phosphate oxidized form
